# Effects of salting treatment on the physicochemical properties, textural properties, and microstructures of duck eggs

**DOI:** 10.1371/journal.pone.0182912

**Published:** 2017-08-10

**Authors:** Lilan Xu, Yan Zhao, Mingsheng Xu, Yao Yao, Xuliang Nie, Huaying Du, Yong-gang Tu

**Affiliations:** 1 Jiangxi Key Laboratory of Natural Products and Functional Food, Jiangxi Agricultural University, Nanchang, China; 2 Engineering Research Center of Biomass Conversion, Ministry of Education, Nanchang University, Nanchang, China; 3 State Key Laboratory of Food Science and Technology, Nanchang University, Nanchang, China; Gaziosmanpasa Universitesi, TURKEY

## Abstract

In order to illuminate the forming process of salted egg, the effects of the brine solution with different salt concentrations on the physicochemical properties, textural properties, and microstructures of duck eggs were evaluated using conventional physicochemical property determination methods. The results showed that the moisture contents of both the raw and cooked egg whites and egg yolks, the springiness of the raw egg yolks and cooked egg whites exhibited a decreasing trend with the increase in the salting time and salt concentration. The salt content, oil exudation and the hardness of the raw egg yolks showed a constantly increasing trend. Viscosity of the raw egg whites showed an overall trend in which it first deceased and then increased and decreased again, which was similar to the trend of the hardness of the cooked egg whites and egg yolks. As the salting proceeded, the pH value of the raw and cooked egg whites declined remarkably and then declined slowly, whereas the pH of the raw and cooked egg yolks did not show any noticeable changes. The effect of salting on the pH value varied significantly with the salt concentration in the brine solution. Scanning electron microscopy (SEM) revealed that salted yolks consist of spherical granules and embedded flattened porosities. It was concluded that the treatment of salt induces solidification of yolk, accompanied with higher oil exudation and the development of a gritty texture. Different salt concentrations show certain differences.

## Introduction

Salted eggs, which are a traditional Chinese egg product, are favored by Chinese consumers’ and have become very popular in other Asian countries due to their unique flavor, attractive taste, and rich nutrient content. Additionally, salted egg yolks are widely used as a material for special cuisine or as a filling material for traditional Chinese foods, such as mooncake, Zongzi (traditional Chinese rice pudding), and egg yolk puffs.

The production of salted eggs typically requires the treatment of fresh duck eggs with a high salt concentration to the develop unique ‘fresh, fine, tender, loose, gritty and oily texture’ features and superior storage properties, which are a result of a series of physicochemical changes [1]. Although the salting process gives the eggs various outstanding features, a large amount of salt is also introduced into the eggs; for instance, the salt content in the egg whites can reach 7%-10% after salting [2]. Excessive sodium intake from a long-term high-salt diet can cause an imbalance in the sodium to potassium ratio and can cause diseases (e.g., hypertension). Moreover, excessive salt intake can lead to edema due to the increased amount of extracellular fluid [3]. Reducing salt consumption and developing salt substitutes have become hot topics in research. Thus, as a major egg product, salted eggs are bound to become an important research subject for reducing salt consumption. Recently, with the boom of the egg product processing industry and the accelerating scale-up of salted egg production, a large amount of research have focused on the changes in the physical and chemical properties of ducks, shortening the pickling cycle and adding additives to improve quality of salted eggs [4–8]. However, no satisfactory breakthroughs have been achieved to improve the processing technology for low-salt egg products. A major reason for the lack of breakthroughs is that the processes and mechanisms involved in the maturation of salted eggs during salting are unclear, which hinders targeted improvement of the processing technology to produce low-salt products.

Therefore, this study investigated the effects of salting treatments in a brine solution for different durations and different salt concentrations and heat treatment on the moisture content, salt content, oil exudation, viscosity, textural properties, and microstructure of salted eggs to provide basic data for further research on egg salting.

## Materials and methods

### Materials

Fresh duck eggs were obtained from a Jiangxi Agricultural University farm in Jiangxi Province, China. Sodium chloride (NaCl), potassium ferrocyanide, potassium chromate, n-hexane, iso- propyl alcohol, disodium hydrogenorthophosphate, silver nitrate, sodium dihydrogen phosphate and ethanol were purchased from Sinopharm Chemical Reagent Co., Ltd. (Shanghai, China). Zinc acetate and glutaraldehyde were purchased from Xilong Chemical CO.,Ltd. (Guangdongi, China) and Jingchun Scientific Co., Ltd. (Shanghai, China), respectively.

### Salting of duck eggs

Fresh duck eggs, less than 3 days after laying, weighing 65 g to 75 g were selected from a farm in Nanchang County, Jiangxi Province, China. The eggs were cleaned with tap water and checked for any crack. The duck eggs were completely immersed in the brine solution (10, 15, 20, and 25% salt) at 25°C temperature cabinet and taken every week during salting up to 5 weeks. The ratio of eggs to the brine solution was about 1:1 (wt/wt).

### Sample preparation

Six eggs from each concentration group were chosen for each week. Three eggs were broken without any special treatment (raw yolk). The other three eggs were heated for 10 min in a water bath, following by cooling in running water (cooked yolk). For each treatment, 3 raw and cooked egg whites and egg yolks were manually separated and pooled as the composite samples, and then the samples were subjected to analyses.

### Determination of moisture and salt contents

Moisture content was determined by drying the samples in a hot-air oven at 100±5°C until constant weight (gravimetric method, Chinese standard GB 5009.3–2010) [9]. Approximately 2g of sample was added in the weighting bottle and taken as M (g), and dried at 105°C until a constant, then weighed and taken as m (g). The moisture contents were calculated as follows: moisture (%) = (M-m)/M.

The salt content was measured using direct titer determination method according to the Determination of Sodium Chloride in Foods, National Food Safety Standard [10]. Samples (3g) were added with 50 mL of hot water 70°C. The mixture was stirred for 15min. The mixed samples were diluted with distilled water into a 200 mL volumetric flask. The mixture was filtered through filter paper. Take 10 mL of the filtrate into the flask using by a pipette, then 30 mL distilled water and 1mL of 5% K_2_CrO_4_ were added to the flask and mixed evenly. The mixture was titrated with the standardized AgNO_3_ until the solution became permanently light red. The salt contents were calculated as follows: salt (%) = 5.8×c×v/m, where v is volume titer of AgNO_3_ (mL), c is the AgNO_3_ concentration in mg/ml, and m is the weight of sample (g).

### Determination of oil exudation

Oil was determined according to the method of Kaewmanee and Fltchern [8, 11] with a slight modification. The sample (approximately 3 g) was homogenized with 25 mL of distilled water using an IK homogenizer (Ultra Turrax homogeniser, IKA T18 digital, IKA Works Guangzhou Co., Ltd., China) at a speed of 10,000 rpm for 30 s. The homogenate was centrifuged at 7,500 g (Anke, Model TGL-20B, Shanghai, China) for 30 min, and 25 mL of organic solution (n-hexane: isopropanol = 3:2, v/v) was added to the supernatant to dissolve the float. The top solvent layer obtained was separated using a separating funnel. After the majority of the solvent was evaporated in a water bath (55°C) and the residue was heated at 105°C until a constant. The obtained residue was weighed, and the free lipid content (%) was calculated.

In another set of tests, approximately 1.5 g of sample was added to 20 mL of organic solvent (n-hexane:isopropanol = 3:2, v/v), followed by homogenization at 10,000 rpm for approximately 1 min. The homogenate was filtered through filter paper and placed in a water bath (55°C) to evaporate the majority of the solvent. Subsequently, the residue was heated at 105°C until a constant, the residue was weighed and taken as total lipid content.

Oil exudation was calculated based on the following formula:

$$\text{Oil exudation}=\frac{\text{Free lipid content}}{\text{Total lipid content}}\times 100\text{\%}$$

### Measurement of the pH value

The pH value was measured according to the analytic methods specified by the Hygiene Standards for Eggs and Egg Products, National Food Safety Standard [12]. The procedures for measuring the pH values of the egg yolk and egg white were the same. The sample was homogenized with distilled water (2:1, w/w). Next, 15 g of homogenate (equal to 10 g of sample) was added and mixed with distilled water until a final volume of 150 mL was obtained. The mixture was filtered through double-layer gauze, and 20 mL of the filtrate was used for the pH measurement with a pH meter (PHS-3C, Shanghai Precision Instruments Co., Ltd., China).

### Determination of the rheological properties

Egg yolk samples after mixing evenly were added in the sample adapter. Egg white samples were directly added in the sample adapter. The rheological properties of the salted egg was measured at room (approximately 25–28°C) using a rheometer (Brookfield, R/S-SST, USA). The measured parameters included a testing duration of 30 s, shear force in the range of 0–300 s^-1^, and 30 testing points. The rotor model was CC40.

### Texture profile analysis (TPA)

The TPA was performed as described by Wei [13]. After the salted eggs were cooked, the yolks were rolled on a filter paper to remove the residual egg white. The cooked egg white with a scalpel to cut into about 1×1×1cm3 cubic block and subjected to TPA analysis, whereas whole egg yolks were used for the TPA. The tests were repeated six times in parallel. A TPA texture analyzer (Stable Micro System, Surrey, England) was used with the following parameters: pre-recording speed of 5.0 mm/s, recording speed of 1.0 mm/s, post-recording speed of 5.0 mm/s, compression ratio of 50%, recoverable time of 5 s, and load on trigger point of 5 g. A P/50 cylindrical probe (50 mm diameter) was used for the TPA of the egg yolk, and a P/36R probe was used for the egg white.

### Environmental scanning electron microscopy (ESEM)

Salted egg yolk samples (approximately 0.5 g) were fixed with 2.5% glutaraldehyde for 2 h and washed by floating in distilled water three times for 15 min per wash. The samples were dehydrated in a graded ethanol series (60%, 70%, 80%, 90%, and 95%) 30 min for each step. The samples were freeze-dried in a freeze-dryer (Alpha1-2, Martin Christ, Germany). After gold coating the samples, the microstructure of the egg yolk was observed using an ESEM (Quanta-200F, FEI, Ltd., The Netherlands) under a low-vacuum mode with an accelerating voltage of 10 kV and magnification ranging from 100× to 5000× [14].

### Data analysis

Except for rheological, TPA and ESEM, the experimental design was a completely random design with three replications. Data were presented as mean values with standard deviations. Statistical analyses were performed with the statistical program DPS. One-way analysis of variance (ANOVA) was carried out, and means comparisons were run by Duncan’s multiple range tests. P<0.05 indicated a significant difference.

## Results and discussion

### Effect of salting on the moisture content in the duck egg yolks and egg whites

**Fig 1:**
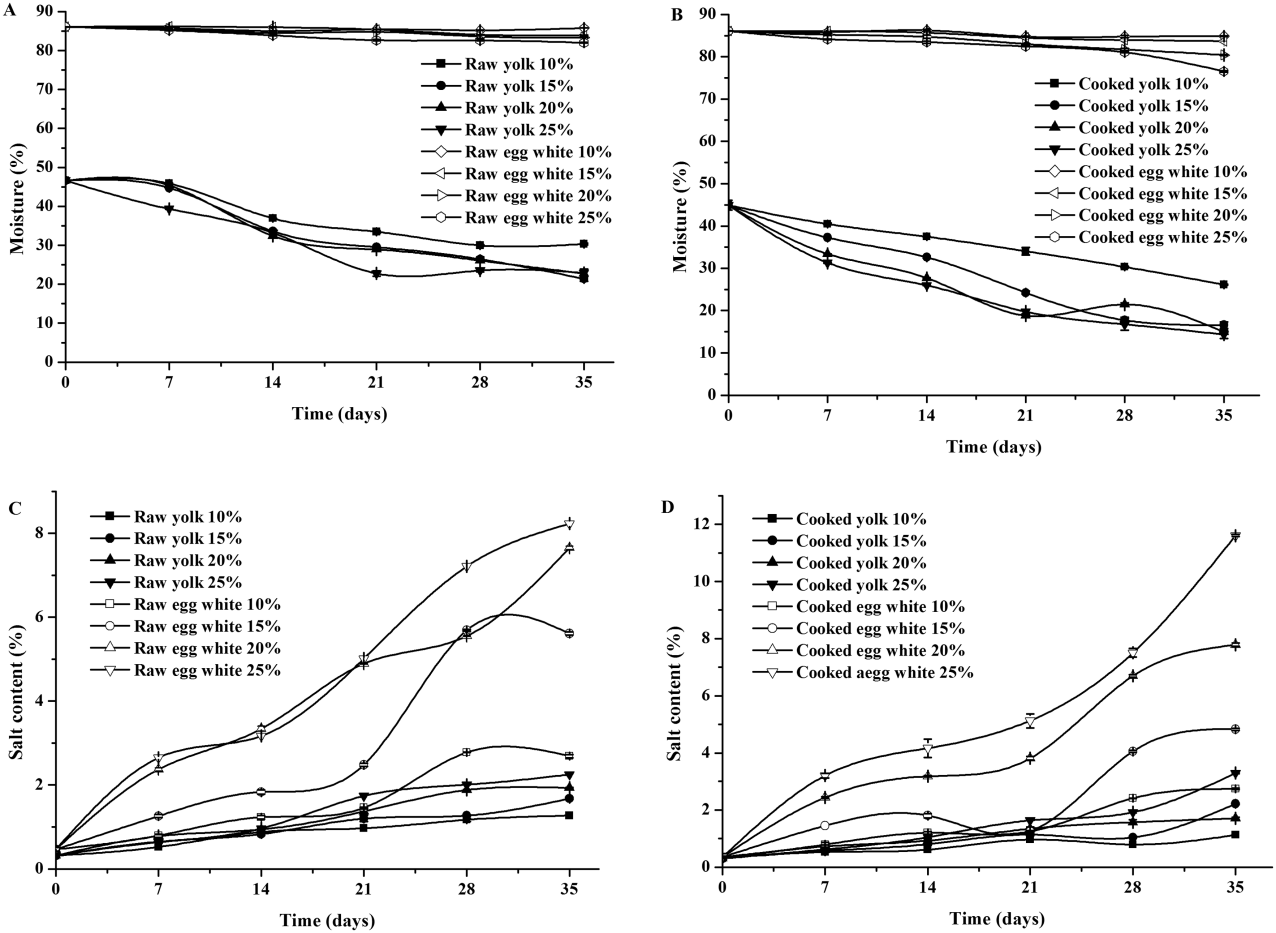
Effects of different concentration of salt on moisture and salt content in the egg yolks and egg whites during salting. (A) The moisture of the raw egg. (B) The moisture of the cooked egg. (C) The salt content of the raw egg. (D) The salt content of the cooked egg.

As shown in Fig 1A and 1B, the moisture contents from fresh duck eggs were 86.07% and 86.05% in the raw and cooked egg whites and 46.65% and 44.91% in the raw and cooked egg yolks, respectively. Increasing the salting time and salt concentration in the brine solution led to a significant reduction in the moisture content in both the egg whites and egg yolks of the raw and cooked salted eggs (P < 0.05). After 35 days of salting, the moisture contents were reduced from 82.01% to 85.82% and from 76.53% to 84.88% in the egg whites and from 22.95% to 30.33% and from 14.36% to 26.14% in the egg yolks of raw and cooked salted eggs, respectively. During salting, the salt molecules diffuse from the egg shell to the egg white and then to the egg yolk, which causes the water to diffuse in a reverse direction from the egg yolk to the egg white and then outside of the egg through the yolk membrane, egg white membrane and egg shell porosities, which ultimately results in a moisture content decline in the egg yolk and egg white [15]. An explanation for the larger moisture decrease in the egg yolk compared to the egg white may be that salting leads to a separation of hydrophilic groups and lipophilic groups in the egg yolk. Consequently, the free water molecules in the egg yolk have relatively increased and diffused to the egg white through the yolk membrane, which results in a larger moisture loss from the egg yolk. With the prolongation of the salting time, the larger moisture decrease in the egg yolks compared to the egg white was similar to the results of kaewmanee and chi [14, 16]. The larger moisture reduction in the eggs salted with a higher concentration of salt may be attributed to the increased osmotic pressure, which accelerates the migration of water molecules.

### Effects of salting on the salt contents in the duck egg yolks and egg whites

During salting, differences in the salt contents lead to an osmotic pressure gradient across the semipermeable membrane system consisting of the inner shell membrane and vitelline membrane of the eggs, which drives the endosmosis of the salt and increases the salt content in the eggs. As shown in Fig 1C and 1D, the salt contents from the fresh duck eggs were 0.47% and 0.35% in the raw and cooked egg whites and 0.32% and 0.30% in the raw and cooked egg yolks, respectively. Increasing the salting time and salt concentration in the brine solution led to a significant increase in the salt content in both the egg whites and egg yolks of the raw and cooked salted eggs (P<0.05). After 35 days of salting, the salt contents increased to maximal levels of 8.23% and 11.61% in the egg whites and 2.25% and 3.29% in the egg yolks of the raw and cooked salted eggs, respectively. The slower salt content change in the salt content of the egg yolk compared to the egg white might have occurred because the salt molecules diffused layer by layer from the outside of the eggs to the egg white through the shell porosities, shell membrane, and egg white membrane before finally reaching the egg yolk. Additionally, the relatively higher lipid content hindered further endosmosis of the salt in the egg yolk.

### Effect of salting on oil exudation in the duck egg yolks

**Fig 2:**
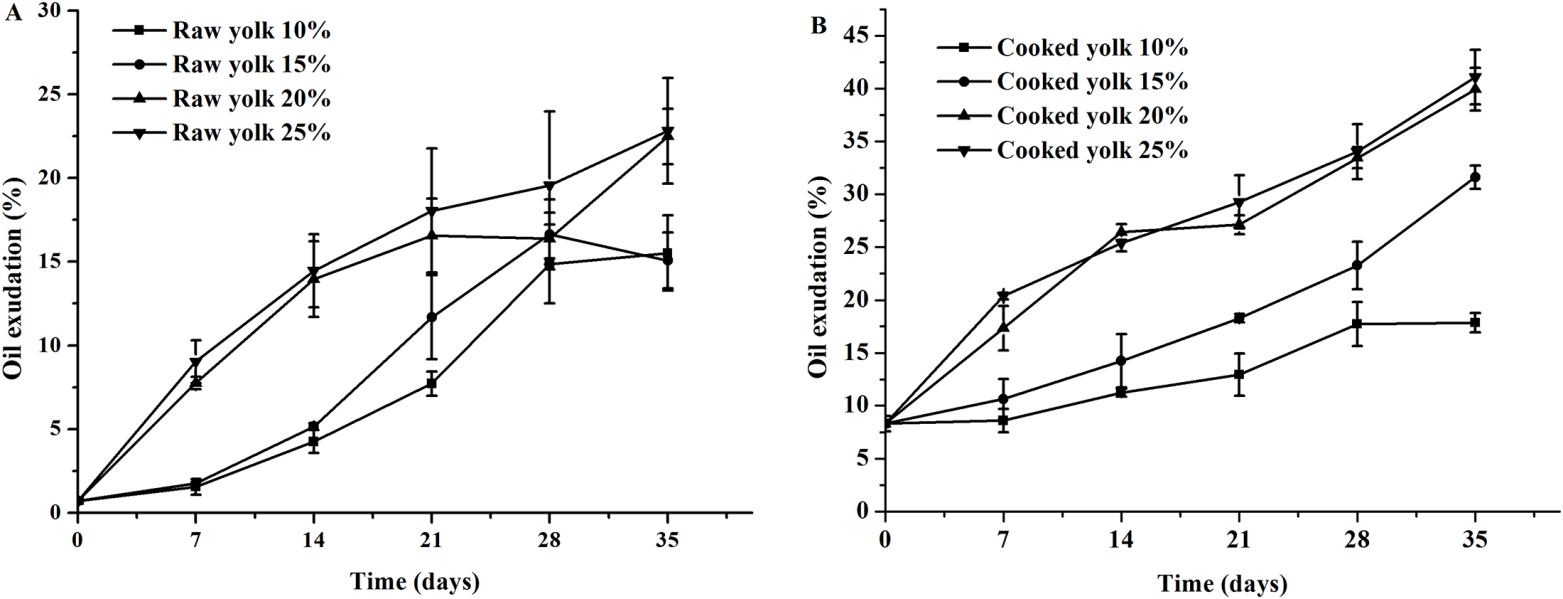
Effects of different concentrations of salt on oil exudation in raw and cooked egg yolks during salting. (A) Raw egg yolk. (B) Cooked egg yolk.

Duck egg yolks are composed of approximately 30–33% lipids. A portion of the low-density lipoproteins lose their emulsification function because of the salt, which results oil exudation during the salting process [17]. As shown in Fig 2A and 2B, the oil exudation of the egg yolk was 0.7% and 8.31% in the raw and cooked fresh duck eggs, respectively, and increased significantly with salting time in the raw and cooked salted eggs (P < 0.05). Additionally, the oil exudation of the egg yolks from the eggs salted with brine solutions containing higher salt concentrations (20% and 25%) increased more than eggs in brine solutions with lower salt concentrations (10% and 15%) (P < 0.05). As the salt concentration in the brine solution increased, the oil exudation of the egg yolk from the cooked salted eggs significantly increased at first (P < 0.05) and then changed insignificantly. After 35 days of salting, the oil exudation of the raw and cooked salted egg yolks increased from 15.51% to 17.85% and from 22.82% to 41.06%, respectively. It possibly because the dehydration effect of the salt enhanced the extraction of oil and resulted in the release of free lipids due to structural changes in the low-density lipoproteins during the salting process [18]. Salting causes a gradual separation and exudation of emulsified fine lipid globules that are originally evenly distributed in the egg yolks, which enhances moisture diffusion towards the outside of the eggs and increases the opportunities for the aggregation of lipophilic groups and the subsequent formation of visible oil liquid or oil droplets. An explanation for the varying effects of the salt concentrations on the oil exudation of the raw and cooked salted egg yolks is that the low concentration salt solution causes massive endosmosis of the salt into the egg yolk through the egg white and thus drastically increases the oil content [5]. However, as the salt concentration increases, the increased oil content in the egg yolks hinders further endosmosis of the salt; ultimately, an equilibrium state of salt endosmosis is achieved in the egg yolks. As a result, the effect of salting on the oil content in the egg yolk does not significantly differ between the high concentration salt solutions. The higher oil exudation of the cooked salted egg yolk compared to the raw salted egg yolk is attributed to the liberation of a portion of the lipids in the egg yolk by the heating treatment [19]. The destruction of lipoproteins during salting might be associated with the shrinkage of yolk granule sizes, whereas heating treatment can further facilitate the separation of the granules [20].

### Effects of salting on the pH values of the duck egg yolks and egg whites

**Fig 3:**
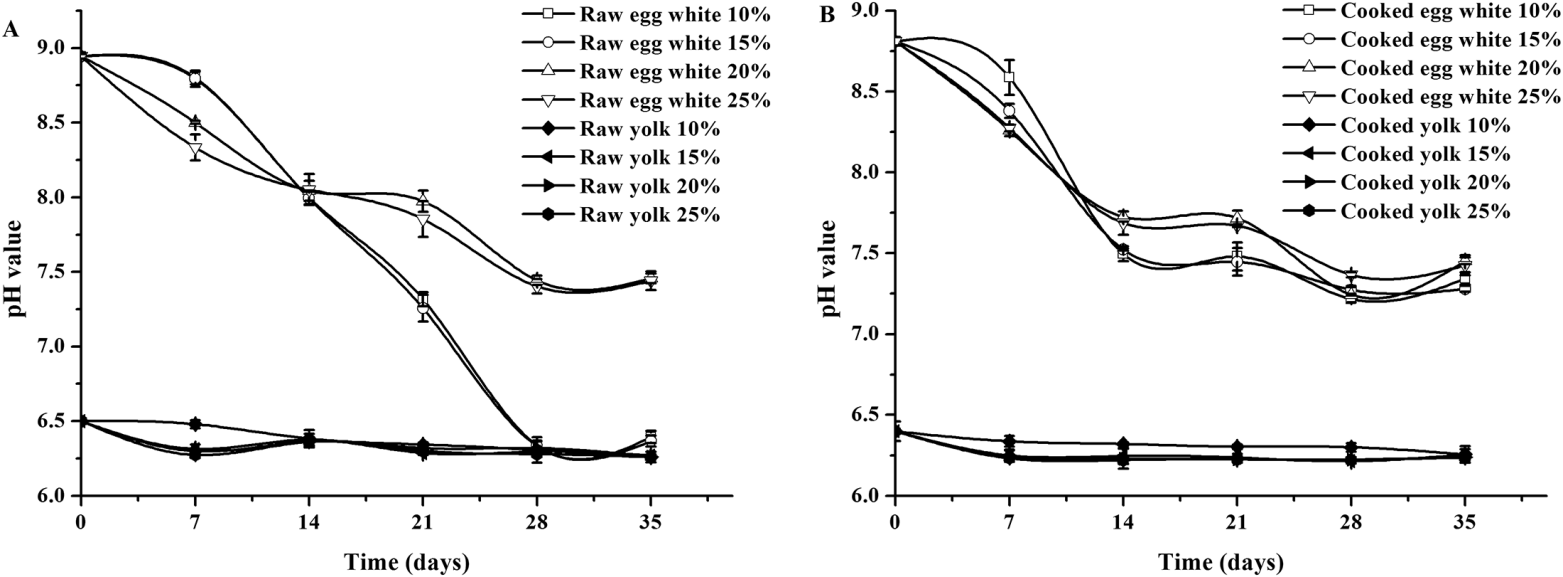
Effects of different concentrations of salt on the pH values in egg yolks and egg whites during salting. (A) Raw egg. (B) Cooked egg.

As shown in Fig 3A and 3B, the pH values were 8.94 and 8.81 for fresh egg white and 6.50 and 6.40 for fresh egg yolks for the raw and cooked salted eggs, respectively. As the salting continued, the pH values of the raw and cooked egg whites showed a trend in which there was a significant decrease (P < 0.05), followed by a basically stable pH during the late stage of salting. However, the change in the pH values varied with the salt concentrations to a certain extent. Higher salt concentrations (20% and 25%) in the brine solution led to higher pH values of the egg white than lower salt concentrations (10% and 15%) (P < 0.05) in both the raw and cooked salted eggs. Moreover, the pH value of the cooked egg yolks decreased significantly after 7 days of salting (P < 0.05); the decrease slowed after this time. The pH values of the raw and cooked egg yolks showed similar decreasing patterns among eggs salted in the 15, 20, and 25% brine solutions but with a greater decrease compared to eggs salted with the 10% brine solution (P < 0.05). After 35 days of salting, the pH values of the raw egg whites, cooked egg whites, raw egg yolks, and cooked egg yolks declined to 6.33, 7.21, 6.26, and 6.21, respectively.

The decreasing pH values of the salted egg whites and egg yolks with increasing salting time might be caused by the destruction of basic proteins (i.e., lysozyme) in the egg white by endosmosis, the reduction of the egg moisture content, the enhancement of the release of carbonic acid gas from the eggs, and the increase in the lipid content in the egg yolk. The higher pH value of the egg whites from the raw and cooked eggs salted with higher concentrations of salt compared to the eggs salted with lower concentrations of salt might be attributed to a reduction of the ovomucin level in the egg white. The pH value is negatively correlated with the ovomucin level in the egg white [21]. The eggs salted in a brine solution containing a higher salt concentration have a higher salt content in the egg white, which dissociates the structure of the ovomucin and causes the egg white to thin and decreases the ovomucin level, leading to an increased pH value in the egg white. Moreover, in the eggs salted with low salt concentrations (10% and 15%), the pH values of the raw egg whites were higher than those of the cooked egg whites after 0–14 days of salting but lower than the cooked eggs after 21–35 days of salting. This phenomenon might be attributed to the varying thermostability of lysozyme under different conditions. During the early stage of the salting process, the egg white is slightly alkaline. Lysozyme has very poor thermostability under alkaline conditions [22]. Consequently, heating might destroy the lysozyme, which leads to a lower pH value of the cooked salted egg whites compared to the raw egg whites. The pH value of the egg white reaches a nearly neutral level during the late stage of salting. The higher pH value of the raw egg whites compared to the cooked egg whites in the eggs salted in higher brine solution concentrations (20% and 25%) might occur because heating causes structural changes of basic proteins (i.e., lysozyme) [23]. Moreover, a high NaCl concentration can induce the thermal aggregation of proteins [24]. As a result, the enhanced protein-protein interactions or interactions between proteins and molecules in the solution result in structural changes in the proteins, which cause higher pH values in the cooked salted egg whites compared to the raw salted egg whites.

### Effects of salting on the rheological properties of the duck egg yolks and egg whites

#### Changes in viscosity of egg whites with shear rate

**Fig 4:**
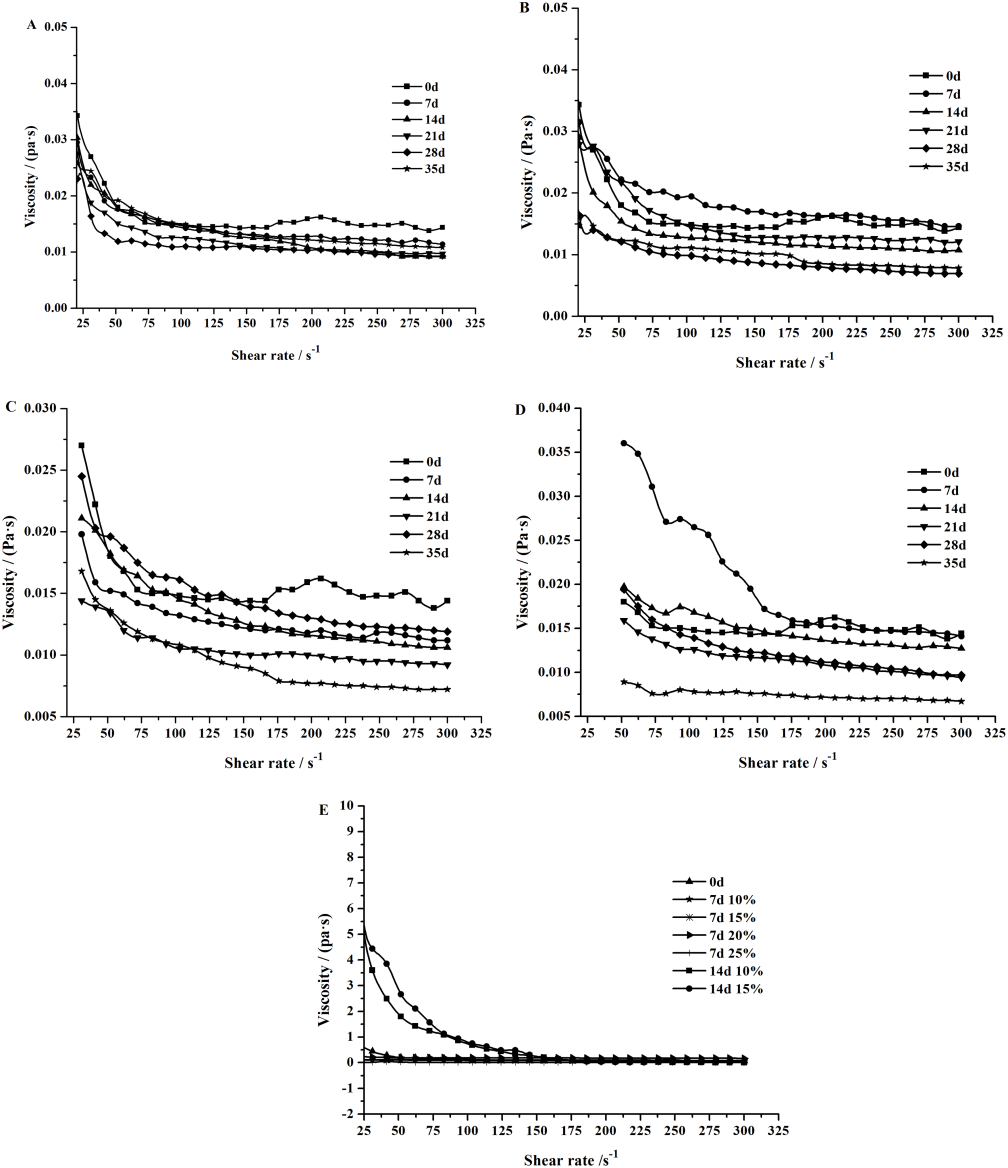
Relationship between the shear rate and viscosity of the raw egg whites and yolks at different stages. (A) Egg white 10% salt. (B) Egg white 15% salt. (C) Egg white 20% salt. (D) Egg white 25% salt. (E) Egg yolk.

Ovomucin is a highly polymerized large molecule protein that is considered to be a key protein in the maintenance of the egg white viscosity [25]. Ovomucin can bind to electrolytes and is very sensitive to salts [26]. Fig 4 present the changing pattern of the egg white viscosity with the salting duration and salt concentration in the brine solution. As salting progresses, the viscosity of the egg white decreases as the shear rate increases, which is a characteristic of shear-thinning fluid. In this study, the egg white viscosity showed an overall decreasing trend after salting, although the viscosity change varied with the salt concentration in the brine solution to a certain extent. In the 10% brine solution, the egg white viscosity slowly declined during the salting period from 0 to 28 days. However, after day 35 of salting, the viscosity of the egg white increased to a level higher than that observed on day 14 of salting. A large number of acidic amino acids results in massive negative charges on the ovomucin. As the salting time increases, the salt content in the egg white might affect the charges carried by the proteins, which reduces the viscosity. After 35 days of salting, the pH value of the egg white was 6.39, which was close to the pH value obtained when ovomucin had the highest molecular weight [27], which lead to an increased viscosity. Therefore, the pH value and viscosity of salted egg whites showed some similarities. In the eggs salted in brine solutions with 15% and 20% salt, the viscosity of the egg white decreased from 0–21 days of salting, then increased until day 28, and finally decreased to the lowest level on day 35. This result might be attributed to the transformation of insoluble ovomucin towards soluble ovomucin and its resulting structural dissociation from ovomucin-lipoprotein complexes, which ultimately leads to a decrease in viscous proteins and the subsequent liquidation and decrease in the viscosity of the egg white [28]. In the eggs salted with the 25% brine solution, the egg white viscosity showed an overall declining trend over time during salting but on day 7, the viscosity was higher than for the fresh egg white. A possible explanation is that the addition of a low concentration of salt enhances the interaction between the proteins and solvent and thereby improves the egg white viscosity [29]. However, we did not find the same phenomenon in the eggs salted with a low-salt brine solution (10%), which might be related to the testing times selected in this study.

#### Changes in viscosity of egg yolks with shear rate

In the eggs salted with the 20% brine solution, the egg yolk began to harden and its viscosity began to increase until it became difficult to detect by the rheometer on day 14 of salting. Thus, the fresh egg yolks, the egg yolks from the eggs salted in the four different brine solution concentrations for seven days, and the egg yolks from the eggs salted in the 10% and 15% brine solutions for 14 days were processed. The rheological properties were measured to obtain the viscosity variation patterns with the egg yolk shear rates.

As shown in Fig 4E, the egg yolk viscosity remained stable on days 0 and 7 of salting even as the shear rate increased. However, the egg yolks from the eggs salted in the 10% and 15% brine solutions for 14 days showed a declining viscosity as the shear rate increased, suggesting that the egg yolk was a shear-thinning fluid at this time. The gradually increasing viscosity of the egg yolks with the increase in the salting time and salt concentration might be attributed to the increase in protein denaturation because of the endosmosis of the salts and dehydration of the egg yolk, which causes the egg yolk to harden and lose flowability. Additionally, the lipid components and bound lipids in a form of emulsified oil droplets in the raw egg yolk affect the rheological behavior of the egg yolk gel [30].

### Effects of salting on the textural properties of the duck egg yolks and egg whites

#### Effects of salting on the textural properties of the raw egg yolks

Because the raw egg yolks began to harden after 14 days of salting in the 20% brine solution, the raw egg yolk samples from eggs salted for 14 days in the 20% and 25% brine solutions and for 21, 28, and 35 days in the brine solutions at all four concentrations were processed to determine the gel hardness and springiness.

**Fig 5:**
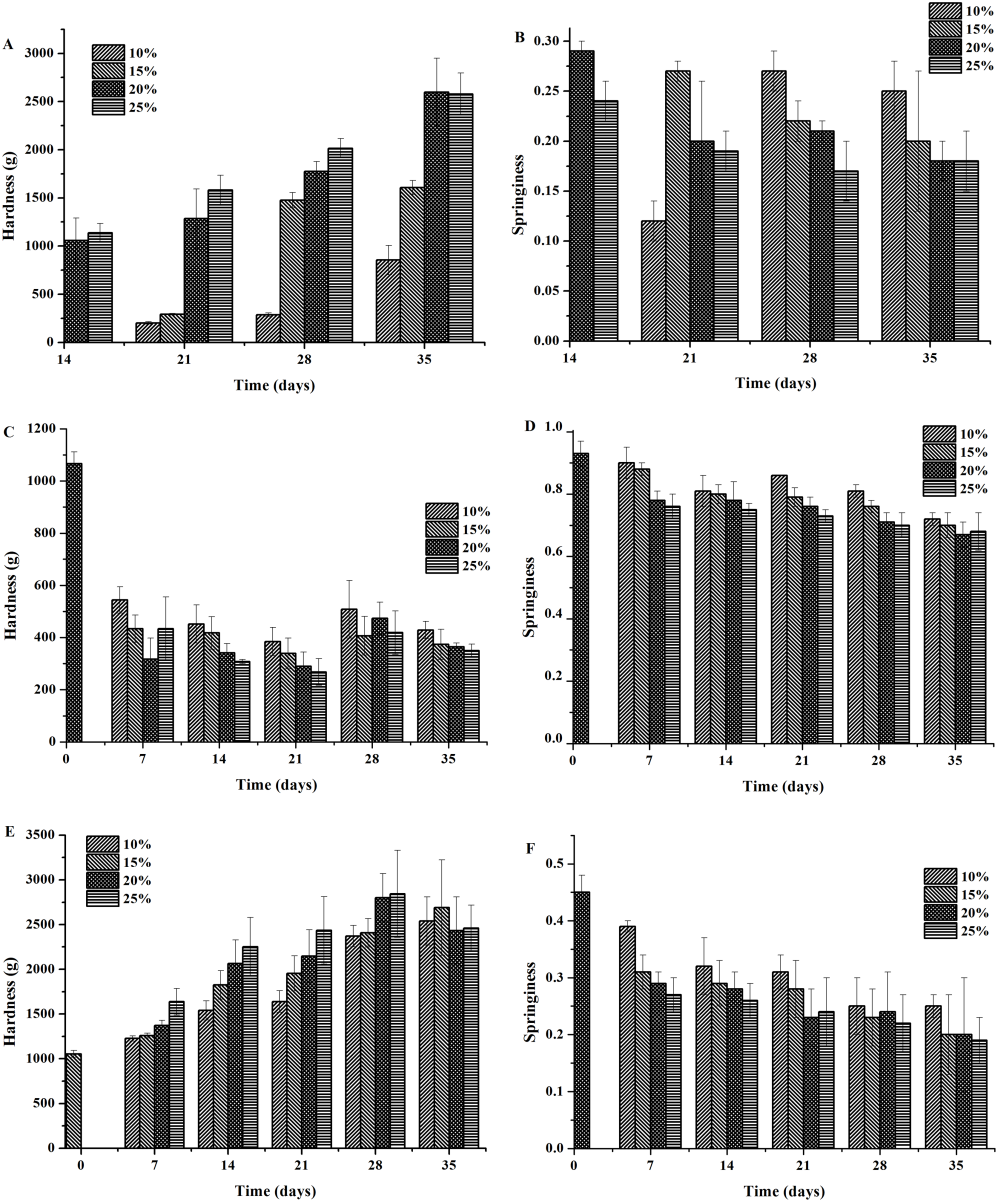
Effects of different concentrations of salt on the hardness and springiness of the raw egg yolks, cooked egg yolks, and egg whites during salting. (A) The hardness of the raw yolks. (B) The springiness of the raw yolks. (C) The hardness of the cooked egg whites. (D) The springiness of the cooked egg whites. (E) The hardness of the cooked yolks. (F) The springiness of the cooked yolks.

As shown in Fig 5A and 5B, the raw salted egg yolks showed an increasing trend of hardness and an overall decreasing trend of springiness as the salting time and salt concentration increased. This result might have occurred because a higher concentration of salt in the brine solution led to a higher degree of protein denaturation, thereby facilitating the formation of a gel network in the egg yolk. The formation of the gel network results from protein denaturation, which enhances the intermolecular interactions and changes the egg yolk into a hardness and springiness structure [31].

#### Effects of salting on the textural properties of the cooked salted egg whites and egg yolks

Fig 5C and 5D shows the changing patterns of hardness and springiness of cooked egg whites as the salting time and salt concentration in the brine solution increase. The hardness of the cooked egg whites decreased over time from 0–21 days of salting, increased on day 28, and then decreased again on day 35 of salting. The cooked egg white from the eggs salted with the 10% brine solution exhibited a higher hardness than the eggs salted with the 15, 20, and 25% brine solutions (P<0.05). The hardness showed a variation pattern similar to the rheological behavior. The springiness of the cooked egg whites displayed an overall decreasing trend as the salting time and salt concentration in the brine solution increased. The decreased springiness and hardness of the cooked egg whites might be caused by the sodium ions directly affecting the stretching of protein molecules and increasing the randomly aggregated components in the gel, which ultimately leads to a coarse texture and uneven gel network in the egg whites. Moreover, metal ions and the pH value also play significant roles in the protein gelation behavior in egg whites. The NaCl content in the egg white increases as the salting time and salt concentration in the brine solution increases, and a high NaCl concentration can decrease the water-retaining capacity of the gel [32]. Moreover, the addition of salt increases the denaturation temperature of the proteins in the solution and delays the aggregation of protein molecules [33], thereby lowering the hardness and springiness of the egg whites. The enhanced hardness observed on day 28 of salting might be attributed to the effect of salt on the electrostatic interactions. Metal cations can shield the negative charges of a protein, thereby decreasing the repulsive force and enhancing the interactions between protein molecules, which promotes molecular aggregation [13, 34] and improves the hardness of the egg whites.

As shown in Fig 5E and 5F, the hardness of the cooked salted egg yolks showed an overall trend of an increase followed by a decrease as the salting time and salt concentration in the brine solution increases. The hardness of the cooked salted egg yolks reached a peak level of 2843.83 g on day 28 in the 25% brine solution but showed a decrease on day 35 in the 20% and 25% brine solutions. A possible explanation for the increasing hardness at the early stage of salting is that the salt content diffusing into the egg yolk does not remarkably affect the aggregation of proteins but instead results in the rupture of egg yolk globules and the release of yolk granules, thereby promoting gelation and enhancing the hardness of the egg yolk [13]. The reason for the decrease in the hardness of the cooked egg yolks at the late stage of salting might be that the yolk globules and yolk granules gradually become smaller and the oil exudation of the egg yolks increases over time during salting; as a result, the oil droplets exuding from the egg yolks act as a lubricant between the egg yolk globules/granules. Finally, the declining springiness of the cooked egg yolks with the increasing salting time and salt concentration in the brine solution can be attributed to the enhancement of protein denaturation by endosmosis and the dehydrating effect of salts, which cause the protein granules to be loosely packed in the egg yolk and thereby reduces the egg yolk springiness.

A comparison of Fig 5A, 5B, 5E and 5F demonstrated that the hardness and springiness of the cooked egg yolks were higher than that of the raw egg yolks (P<0.05), possibly because even a slight disturbance of the raw fresh egg yolks can cause 90–95% of the granules to rupture and release their contents. Thus, rolling an intact fresh egg yolk on a piece of coarse cloth might destroy the egg yolk globules and improve its post-heating gelation behavior [35]. The comparisons between Fig 5C and 5D and between Fig 5E and 5F showed that the cooked egg yolk gel had a hardness that was much higher than the cooked egg white gel (P<0.05) during the entire salting process, the springiness of the yolks gel was constantly lower than that of the latter (P<0.05). In addition to water, another major component of egg whites is highly diverse proteins. These proteins have complex structures that crosslink with one another under the effects of salt. Heating can damage the tertiary and quaternary structures of the egg white proteins and enhance the interactions between protein molecules or between proteins and molecules in the solution. As a result, the α-helices are reduced and the β-folds are increased in the secondary structures of the ovalbumin, ovotransferrin, and lysozyme. Subsequently, the increase in β-fold structures causes the three-dimensional protein network structures to be denser and thereby improves the hardness of the gel [23]. When the pH value is low, insoluble polymers exist between the protein molecules in the egg yolk [36]. When the pH value becomes closer to the isoelectric point of the low-density lipoprotein in the egg yolk after salting, heating treatment can cause the protein molecules to aggregate rapidly because of the decreasing repulsive forces between the protein molecules. However, a large number of polymers that might exist in the formed gel network and a large number of lipids in the salted egg yolk can reduce the springiness of the gel.

### Effect of salting on the egg yolk microstructures

**Fig 6:**
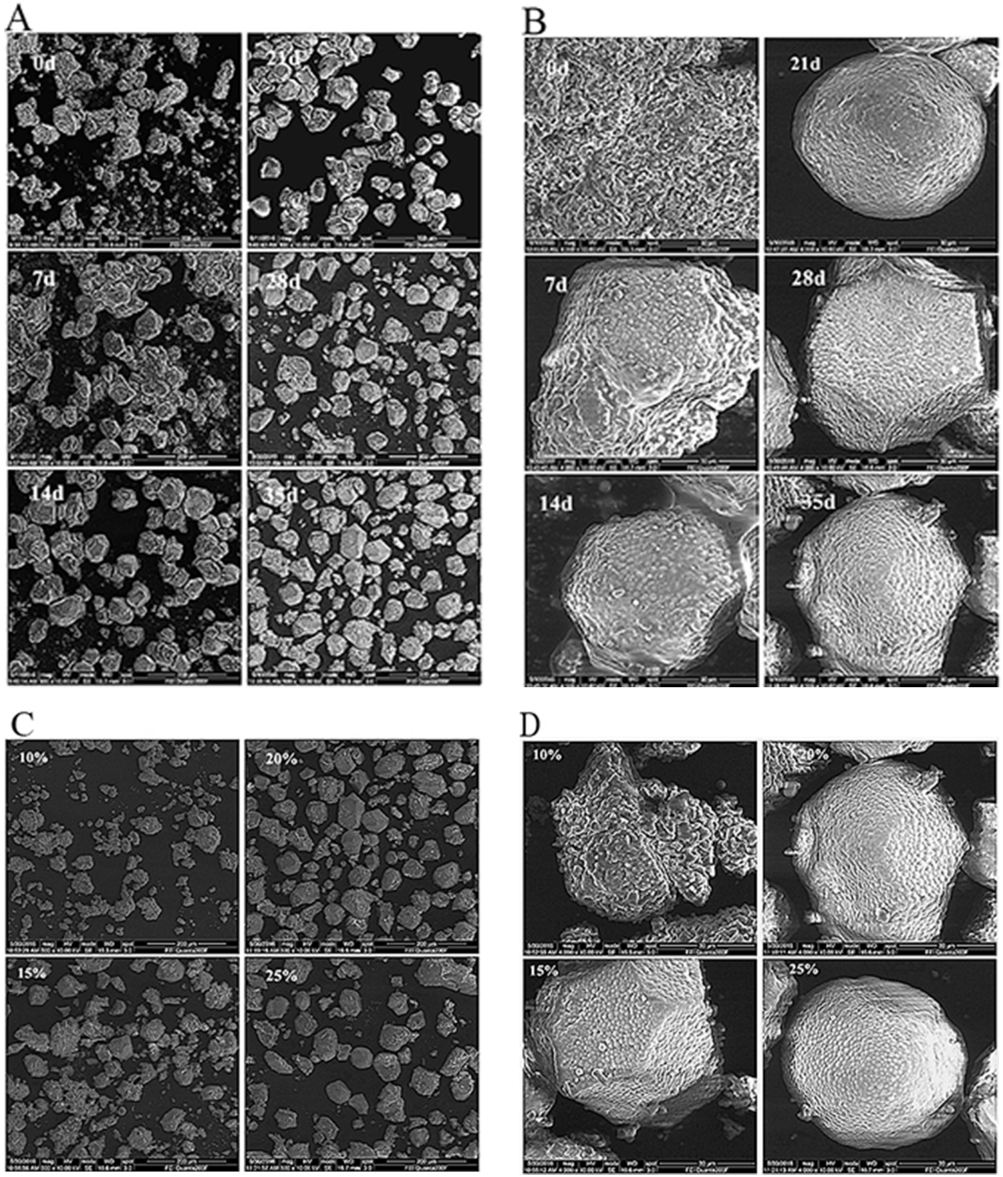
Effect of salting on the egg yolk microstructures. Scanning electron micrograph (A, B) of cooked salted egg yolks in a 20% NaCl solution during salting. Micrographs (C, D) of the salted egg yolks in the four different brine solution concentrations for 35 days. Lower magnification (A, C): 500×f and higher magnification (B, D): 4000×f pictures.

As shown in Fig 6A and 6B, the surface of the egg yolk globules consists of spherical granules (0.5–3.0 μm in diameter) and embedded flattened porosities surrounding the spherical granules. Yolk globules adopt various polyhedral shapes, and there is no adhesion between yolk globules. The morphological changes in yolk globules might result from the sample fixation process [37]. Yolk globules can be roughly identified according to their diameters, which range between 0.2 μm and 2 μm [38]. Bellairs et al. reported that these globules were granular and were actually lipoprotein micro-droplets [39]. After salting for 0 to 7 days, the individual yolk globules could not be observed because they were wrapped in a layer of slimy substances, which are the result of the polar and non-polar lipid molecules gathering together [30]. With an increase in the salting time, the yolk globules with clear contours were observed during the later stage of salting, and the size of the yolk globules decreased from 52.25 to 46.75 μm. In addition to the size decrease of the yolk globules and yolk granules, the yolk globules were more densely and evenly distributed and the number of flattened porosities embedded between the yolk granules was reduced. Moreover, the yolk granules were more densely packed and an increased number of yolk granules fell off from the yolk globules. Previous research found that the size of the yolk granules in salted eggs ranged from 90–100 μm and 30–75 μm [40]. An explanation for the more even and denser distribution of yolk globules may be that the enhanced dehydration of the egg yolk with the salting time leads to an enhancement of the links between the yolk globules. The increasing number of yolk granules that fall off of the yolk globules might be associated with the ionic strength of NaCl in the egg yolk. Yolk granules primarily consist of high-density lipoprotein-phosvitin complexes formed via calcium-phosphorus bridges. As the salting time increases, the increased salt content in the egg yolk can cause the replacement of Ca^2+^ by Na^+^ in the calcium-phosphorus bridges that maintain the yolk granule structures, thereby resulting in a destruction of the egg yolk granules and the subsequent dissolution of soluble phosvitin [41].

As shown in Fig 6C and 6D, as the sizes of the egg yolk globules/granules decreased, the aggregation of the yolk granules became more noticeable, an increasing number of granular proteins were released from the yolk globules, and no cross-links were observed between the yolk globules in the salted eggs as the salt concentration increased. The size decrease in the yolk granules during salting might be related to the destruction of lipoproteins. The increasing number of yolk falling off of the yolk globules might be associated with oil exudation of the egg yolk. The shrinkage and even aggregation of egg yolk globules/granules might contribute to the unique gritty texture of the egg yolk [42]. The egg yolk globules wrapped by the semipermeable membrane swell and explode in the low-concentration solution and shrink in the high-concentration solution. The egg yolk globules can release protein granules after their surface membrane expands in a filament pattern. Our results revealed a relatively large impact of the salting time and salt concentration in the brine solution on the microstructures of salted egg yolks.

## Conclusions

Salting can substantially change the physicochemical properties, textural properties and microstructures of duck eggs, resulting in a series of unique qualities. As salting proceeds, salt endosmosis induces gelation and hardening of the egg yolk, which causes a decrease in the moisture content and pH value and an increase in the salt content, oil exudation, hardness, and viscosity. Additionally, the yolk granules are gradually destroyed, and the yolk globules become evenly and densely distributed. Salting results in liquidation of the egg white, leading to a declining moisture content, pH value, and viscosity and increasing salt content and hardness. The changes in the egg whites and egg yolks of duck eggs during salting involve different mechanisms. The effects of salting on the physicochemical properties, textural properties and microstructures of duck eggs vary with the salt concentration in the brine solution to a certain extent. Higher salt concentrations in the brine solution lead to higher pH values but lower springiness and viscosity of the raw egg whites. Heating treatment can lower the moisture content in both the egg yolks and egg whites, increase the oil exudation and hardiness of the egg yolks, and decrease the springiness and pH value of the egg yolks. Based on this study, 20% brine solutions and 28–35 days of salting at 25°C is the appropriate processing conditions of salted egg. The forming mechanism of unique characteristic of salted egg will be studied in our future work.

## Supporting information

S1 File Effects of salting on the moisture and salt content in the duck egg yolks and egg whites. (XLSX)

S2 File Effects of salting on oil exudation in the duck egg yolks. (XLSX)

S3 File Effects of salting on pH values of the duck egg yolks and egg whites. (XLSX)

S4 File Effects of salting on the rheological properties of the duck egg yolks and egg whites. (XLSX)

S5 File Effects of salting on the textural properties of the duck egg yolks and egg whites. (XLSX)
